# The effectiveness of extracorporeal shock wave therapy for the treatment of lower limb ulceration: a systematic review

**DOI:** 10.1186/s13047-014-0059-0

**Published:** 2015-02-05

**Authors:** Paul A Butterworth, Tom P Walsh, Yvonne D Pennisi, Anna D Chesne, Christoph Schmitz, Susan A Nancarrow

**Affiliations:** School of Health and Human Sciences, Southern Cross University, ᅟ, Queensland 4225 Australia; Lower Extremity and Gait Studies Program, Faculty of Health Sciences, La Trobe University, Melbourne, Victoria 3086 Australia; School of Medicine, Faculty of Medicine, Nursing and Health Science, Flinders University South Australia, Adelaide, 5042 Australia; Department of Anatomy II, Ludwig-Maximilians-University of Munich, Munich, 80336 Germany

## Abstract

Extracorporeal shock wave therapy has been reported as an effective treatment for lower limb ulceration. The aim of this systematic review was to investigate the effectiveness of extracorporeal shock wave therapy for the treatment of lower limb ulceration.

Five electronic databases (Ovid MEDLINE, CINAHL, Web of Knowledge, Scopus and Ovid AMED) and reference lists from relevant studies were searched in December 2013. All study designs, with the exception of case-reports, were eligible for inclusion in this review. Assessment of each study’s methodological quality was performed using the Quality Index tool. The effectiveness of studies was measured by calculating effect sizes (Cohen’s *d*) from means and standard deviations.

Five studies, including; three randomised controlled trials, one quasi-experimental study and one case-series design met our inclusion criteria and were reviewed. Quality assessment scores ranged from 38 to 63% (mean 53%). Improvements in wound healing were identified in these studies following extracorporeal shock wave therapy. The majority of wounds assessed were associated with diabetes and the effectiveness of ESWT as an addition to standard care has only been assessed in one randomised controlled trial.

Considering the limited evidence identified, further research is needed to support the use of extracorporeal shock wave therapy in the treatment of lower limb ulceration.

**Electronic supplementary material:**

The online version of this article (doi:10.1186/s13047-014-0059-0) contains supplementary material, which is available to authorized users.

## Introduction

Lower limb ulceration is reported as a common problem world-wide, and is considered a major social and economic burden [1]. Lower limb ulceration is associated with numerous comorbidities including, but not limited to; diabetes, peripheral vascular disease and venous insufficiency [2]. The management of ulceration is dependent on the proposed causes, although common interventions may include both non-surgical and surgical approaches [3]. Typically, effective ulcer management involves local wound care, compression therapy, pressure redistribution, infection management and optimization of vascular status [2,4,5]. Recently, extracorporeal shock wave therapy, for the treatment of chronic ulceration, has also gained attention in the literature [6].

The use of extracorporeal shock waves in medicine was first reported over 30 years ago as a treatment for kidney stones [7], and is commonly referred to as ‘extracorporeal shock wave lithotripsy’, or ‘ESWL’ [8]. Extracorporeal shock waves are also used as a treatment for musculoskeletal conditions such as plantar heel pain [9,10] and boney non-union [11,12], and is commonly referred to as ‘extracorporeal shock wave therapy’ (ESWT) to differentiate from ESWL [13]. Furthermore, the use of ESWT has also been reported in the treatment of arterial aneurysms [14] and intermittent claudication [15,16]. Although the mechanisms by which ESWT improves ulcer healing are not fully understood, it is purported to stimulate vascular in-growth, neovascularization and cell proliferation [17,18], therefore improving healing rates in chronic ulcers [1]. Typically, ESWT would entail one to two treatments per week, until there is evidence of ulcer healing.

Despite the reported success of ESWT for the treatment of lower limb ulceration, the quality of evidence investigating the effectiveness of this intervention has not been reviewed in detail. Therefore, the aim of this review was to investigate the effectiveness of ESWT for the treatment of lower limb ulceration.

## Review

### Types of studies included

All studies included in this review were obtained from English-language peer reviewed scientific journals investigating the effectiveness of ESWT for lower limb ulceration. All study designs, with the exception of case-reports, were eligible for inclusion in this review. Letters to the editor, opinion pieces and editorials were also excluded.

### Types of participants included

Studies were included if the use of ESWT for the treatment of lower limb ulceration was assessed. The category of ulcers included in this review were those of neurovascular origin (i.e. diabetic, neuropathic, neurovascular or vascular). Studies where the participant’s ulcer was associated with pressure sores, burns or surgical complications were excluded.

### Search strategy for identification of studies

In December 2013 an electronic database search was conducted using Medical Subject Headings (MeSH), followed by a keyword search strategy. Auto-alerts were developed to provide updates on recent publications until the review was finalised (March 2014). The following databases were searched: Ovid MEDLINE (1966 to date), CINAHL (1982 to date), Web of Knowledge, Scopus and Ovid AMED (from inception). The database search strategy is presented in Table 1.

**Table 1:** **Database search strategy**

Subject term keywords	1. Exp. Extra corporeal shock wave therapy
	2. “Extra corporeal shock wave therapy” or ESWT or “shock wave therapy” or lithotripsy
	3. 1 or 2
Subject term keywords	4. Exp. ulcer
	5. Lower limb or foot* or leg or arterial or venous or neuropathic or diabet*
	6. 4 or 5
Combine	10. 3 and 6

Upon completion of the search (March 2014), a hand search was performed of references from the studies identified in the electronic search, and Google Scholar was searched in an attempt to identify any further material. Two reviewers (PAB and TPW) then independently reviewed titles and abstracts according to the pre-determined inclusion criteria. Discrepancies between reviewers regarding eligibility were discussed until consensus was reached. Progression to full text review was then permitted.

### Data extraction and analysis

A predefined data extraction form was used in the extraction process (Additional file 1). Relevant data (means, mean differences, standard deviations and *p* values) were extracted from studies by two investigators (PAB and TPW), with specific attention to the following variables; study design, participant numbers, mean age, sex, ulcer classification, change in healing and ulcer size and ESWT protocol used. The data pertaining to each study was then assigned a numerical value to ensure the two investigators (PAB and TPW) were blinded to author and publication details during quality assessment. Where disagreements occurred during the quality assessment process, a third assessor (YDP) made the final decision on quality assessment scores. Where studies provided sufficient statistical data, effect size (Cohen’s *d*) was calculated from means and standard deviations. Effect sizes were categorized as follows: negligible effect (≥ − 0.15 and <0.15); small effect (≥0.15 and <0.40); medium effect (≥0.40 and <0.75); large effect (≥0.75 and <1.10); very large effect (≥1.10 and <1.45) and, huge effect (≥1.45) [19,20].

### Assessment of methodological quality

Assessment of each study’s methodological quality was performed using the Quality Index tool developed by Downs and Black [21]. This tool has been shown to have high internal consistency (KR-20 = 0.89), good test-retest reliability (*r* = 0.88) and good inter-rater reliability (*r* = 0.75). The Quality Index tool consists of 27 items, and allows for assessment of internal and external validity, reporting and power.

For this systematic review, an *a priori* decision was made to remove two items where they did not apply to the respective studies identified. Firstly, Item 25 was removed for non-randomised controlled studies, as it has been shown that case mix adjustment cannot reduce the extent of bias in non-randomised trials [22]. Secondly, Item 27 was removed for all studies, as a minimally important difference has not been established for measuring the outcomes of ESWT in the treatment of ulceration.

We chose to present the quality assessment results as percentage scores, which is typical of previous studies using the Quality Index tool [20,23-24]. Furthermore, after obtaining data from the included studies, the findings were combined using a narrative rather than a quantitative approach, owing to study heterogeneity.

### Results

**Figure 1:**
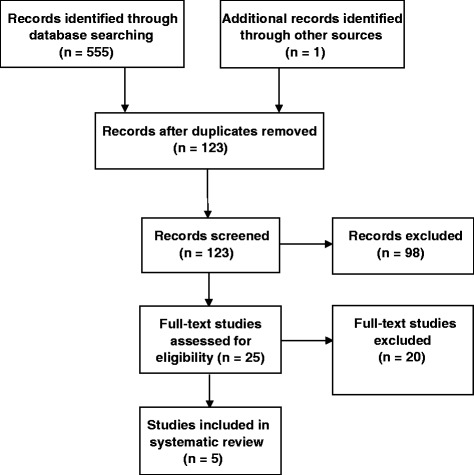
**Study selection process.**

A total of 555 results were identified through our electronic search (Additional file 2) and one further study from other sources (i.e. as laid out in our search strategy). Upon the removal of duplicates, 123 studies were suitable for initial review. Following the review of titles and abstracts, 25 studies were extracted for full review and finally, five studies were considered appropriate for inclusion (Additional file 3). The reasons for the exclusion of studies are available in Additional file 4. A flow diagram, as described by Moher and colleagues [25], is presented in Figure 1, highlighting the study selection process.

### Quality of the evidence

The inter-rater reliability of the Quality Index scores was not calculated due to the small number of trials included in the review [23]. However, perfect agreement was recorded on all items except item 5 (50% agreement) and item 13 (65% agreement). Table 2 indicates that moderate study quality was identified across trials (quality assessment scores ranged from 38 to 63%, mean 53%). External validity across studies was rated most poorly, due to deficient definitions of the source population and methods of patient selection, and poor identification of confounding factors. Studies also rated poorly on the internal validity component of the Quality Index.

**Table 2:** **Quality assessment scores from the Quality index tool** [21]

**Quality index items**	**Moretti** [26]	**Saggini** [27]	**Wang** [28]	**Wang** [29]	**Schaden** [17]
**Reporting**					
1. Study hypotheses/aim/objective	1	0	1	1	1
2. Main outcomes	1	1	1	1	1
3. Participant characteristics	1	1	1	1	1
4. Interventions of interest	1	0	1	1	1
5. Distribution of principal confounders	1	1	0	1	1
6. Main findings	1	1	1	1	1
7. Estimates of random variability	1	0	1	0	1
8. Adverse events described	0	1	0	1	1
9. Participants lost to follow up described	1	0	1	1	1
10. Actual probability values reported	0	0	0	1	1
**External validity**					
11. Were subjects asked to participate representative of population from which they were recruited?	0	0	0	0	0
12. Were subjects prepared to participate representative of the entire population from which they were recruited?	0	0	0	0	0
13. Were the staff, places and facilities where the patients were treated, representative of the treatment patients received?	1	1	0	1	1
**Internal validity (bias)**					
14. Was an attempt made to blind study subjects to the intervention they have received?	0	0	0	0	0
15. Was an attempt made to blind those measuring the main outcomes of the intervention?	0	0	0	0	0
16. If any of the results of the study were based on ‘data dredging’ was this made clear?	1	1	1	0	1
17. Does analysis adjust for lengths of follow up or is the time period between intervention and outcome the same?	0	0	0	0	0
18. Were the statistical tests used to assess the main outcomes appropriate?	0	1	0	1	1
19. Was compliance with the intervention reliable?	1	1	1	1	1
20. Were the main outcome measures used accurate (valid and reliable)?	1	0	1	1	0
**Internal validity (selection bias)**					
21. Were cases and controls recruited from the same population?	0	0	1	1	0
22. Were cases and controls recruited over the same period of time?	1	1	1	0	1
23. Were study subjects randomised to intervention groups?	1	0	1	1	0
24. Was randomised intervention assignment concealed from participants/researchers until recruitment complete?	0	0	0	0	0
25. Was there adequate adjustment for confounding in the analysis from which the main findings were drawn?	0	*	0	0	1
26. Were losses to follow up of patients taken into account?	1	0	1	1	1
**Power**					
27. Did the study have sufficient power to detect a clinically important effect?	*	*	*	*	*
**Total score%**	*55*	*38*	*52*	*59*	*63*

Notes:

All questions were scored on the following scale: yes = 1, unable to determine = 0, no = 0.

Question 5 is an exception with scores allocated: yes = 2, partially = 1, no = 0.

*Item removed.

### Trial characteristics

There were three randomised control trials, one quasi-experimental study and one case-series study identified, the characteristics of which are presented in Table 3. Moretti *et al.* [26] conducted a randomised controlled study in participants with neuropathic diabetic foot ulcers, to evaluate standard care and ESWT (intervention group) against standard care alone (control group). Both groups received standard care consisting of therapeutic footwear, debridement and dressings, although none of these variables were described in detail. Furthermore, the treatment of infection was undertaken where necessary and was not considered as a reason to exclude participants.

**Table 3:** **Characteristics of included studies**

**Primary author**	**Outcomes measured**	**Study design**	**Number allocated to (a) experimental and (b) control groups**	**Mean age years (SD)**	**% female**	**Mean duration of disease in months (SD)**	**Ulcer classification**	**ESWT protocol**
Moretti [26]	Rate of re-epithelialization	RCT	(a) 15	56.2 ± 4.9	40	UD	Neuropathic (diabetic) plantar foot ulceration ≥6 months duration; area >1 cm^2^ and diameter between 0.5 and 5 cm	3 sessions (every 72 hours); 100 pulses per 1 cm^2^; EFD 0.03 mJ/mm^2^
			15	56.8 ± 7.5	53	UD		
Saggini [27]	Exudate,	Quasi-experimental	(a) 30	58.5	43	5.3	Venous ulcers; diabetic ulcers; unresponsive to conservative care for ≥3 months duration	4 to 10 sessions; 100 impulses per 1 cm^2^; EFD 0.037 mJ/mm^2^; frequency of 4 Hz or 240 impulses/min
	Granulation and		10	66.6	40	5.2		
	Fibrin/necrotic tissue							
Wang [28]	Healing rates	RCT	(a) 34	58.6 ± 12.6	UD	22.7 ± 20.9	Diabetic foot ulcer >3 months duration	3 treatments; repeat course performed in cases with incomplete healing; 300 plus 100 pulses per 1 cm^2^; EFD 0.11 mJ/cm^2^
	Histopathological analysis		(a) 36	63.4 ± 10.3	UD	19.0 ± 19.5		
Wang [29]	Healing rates	RCT	(a) 39	60.5 ± 14.0	UD	6* (3 to16)	Diabetic foot ulcer >3 months duration	6 treatments; Ulcer size dependent treatment; minimum 500 pulses; EFD 0.27 mJ/cm^2^
	Histopathological analysis		(a) 38	62.5 ± 14.0	UD	6* (6 to10)		
Schaden [17]	Safety and feasibility of ESWT	Case-series	(a) 31	61.0	48	UD	Complicated, non-healing, acute and chronic venous and arterial ulcers	Mean sessions 1.9 to 3.7; 100 impulses per 1 cm^2^; EFD 0.1 mJ/mm^2^

Notes:

EFD: energy flux density; RCT: randomised controlled trial.

SD: standard deviation; UD: unable to determine.

*Median value (range) reported.

ESWT = extracorporeal shock wave therapy.

Saggini *et al.* [27] conducted a quasi-experimental study and investigated 40 participants with chronic posttraumatic, venous and diabetic ulcers (17 venous ulcers, 7 diabetic). The case group (n = 30) received ESWT; between ESWT treatment sessions, the participants continued previous conservative treatment, although it is unclear what this treatment consisted of. There were 10 control participants who received regular conservative dressings, although the authors did not explain what conservative care involved.

Wang *et al.* [28] compared ESWT with hyperbaric oxygen therapy (HBO) in chronic diabetic foot ulcers. Seventy-four participants were randomly divided into two groups according to the dates that they were referred into the study. Thirty-six participants with 38 ulcers received ESWT, whereas 38 participants with 38 ulcers received HBO therapy. A repeat course of ESWT was performed in cases with incomplete healing from the first course of treatment. Participants resumed the same wound care technique at home after treatment including offloading on the affected leg, wound cleansing with sterile normal saline solution, and application of silver sulfadiazine cream. Participants in the HBO group received the same wound care as the ESWT group. In their second study comparing ESWT with HBO therapy in chronic diabetic foot ulcers, Wang *et al.* [29] randomly divided participants according to computer generated block labels. Forty-three participants were assigned to the ESWT group and 45 participants were assigned to the HBO group. After ESWT, participants resumed their initial wound care protocol including offloading on the affected foot, wound cleansing with sterile normal saline solution and application of silver sulfadiazine cream. Participants in the HBO group received the same wound care protocol after treatment as the ESWT group.

In a study by Schaden *et al.* [17], the safety and feasibility of ESWT was assessed in 208 participants with a variety of wounds that included 31 ulcers of a neurovascular origin. The intervention applied to all participants was a combination of debridement, ESWT and moist wound dressings.

### Evidence for the effectiveness of ESWT in the treatment of lower limb ulceration

Table 4 provides a description of the mean differences of ulcer healing between groups. Moretti *et al.* [26] found that after 20 weeks of treatment, 53.33% of the ESWT group had complete wound closure compared with 33.33% of the control group, and healing times were 60.8 and 82.2 days respectively (*p* < 0.001).

**Table 4:** **Mean differences in ulcer healing between groups of included studies**

**Primary author**	**Difference between groups**	**Control**	***p*** **value**	**Effect size (Cohen’s** ***d*** **)**
	**ESWT**			
Moretti [26]	Wound closure: 53.33%	33.33%	< 0.001	UD
	healing time: 60.8 days (SD 4.7 days)	82.2 days (SD 4.7 days)	<0.001	4.43, Huge effect
Saggini [27]	UD	UD	UD	UD
Wang [28]	31% completely healed	22% completely healed	<0.001	UD
	58% improved	50% improved	<0.001	UD
	11% remained unchanged.	28% remained unchanged	<0.001	UD
	≥50% improved in 89% of participants	≥50% improved in 72% of participants	<0.001	UD
Wang [29]	Completely healed 57%	Completely healed 25%	=0.003	UD
	≥50% improved in 32% of participants	≥50% improved in 15% of participants	=0.071	UD
	unchanged ulcers in 11%	unchanged ulcers in 60%	<0.001	UD
Schaden [17]	NA	NA	NA	NA

Notes:

UD: unable to determine; NA: not applicable.

ESWT = extracorporeal shock wave therapy.

SD = standard deviation.

Cohen’s *d*: negligible effect (≥ − 0.15 and <0.15), small effect (≥0.15 and <0.40), medium effect (≥0.40 and <0.75), large effect (≥0.75 and <1.10), very large effect (≥1.10 and <1.45), huge effect (≥1.45).

Saggini *et al.* [27] treated 32 ulcers with ESWT and reported that 16 ulcers healed completely within six sessions of ESWT. In those ulcers that did not completely heal, statistical significance (p < 0.01) was reported with regard to decrease in ulcer size, after four to six sessions of ESWT. There was no evidence in this study of a difference between the two groups regarding ulcer healing or change in ulcer size.

Wang *et al.* [28] found that in the ESWT group, 31% of ulcers completely healed, 58% improved and 11% remained unchanged. In the HBO group 22% completely healed, 50% improved, and 28% remained unchanged. These differences were significant at *p* = 0.001. Furthermore, greater than 50% improvement of the ulcer was observed in 89% of participants in the ESWT group and 72% of participants in the HBO group (*p* < 0.001). In their second study comparing ESWT and HBO, Wang *et al.* [29] found: completely healed ulcers in 57% and 25% (*p* = 0.003); ≥ 50% improved ulcers in 32% and 15% (*p* = 0.071), and unchanged ulcers in 11% and 60% (*p* < 0.001) respectively.

Schaden *et al.* [17] found that venous stasis ulcers demonstrated the worst healing rates (36% versus 66% for all other ulcers, *p* = 0.001). Furthermore, arterial insufficiency ulcers did not completely heal in 33% of cases, the second worst healing rate of all ulcer types. The primary outcome assessed in their study was the safety and feasibility of using ESWT on wounds, the authors concluding that ESWT is a safe and effective treatment.

### Discussion

The aim of this systematic review was to investigate the effectiveness of ESWT for the treatment of lower limb ulcers. We evaluated five studies in this review, and identified a trend to suggest that ESWT may be effective in improving wound healing and decreasing wound size. Furthermore, ESWT may also be a safe treatment option with few complications associated with its use, however; we found average study quality for the studies identified. External validity across studies was rated most poorly, due to deficient definitions of the source population and methods of patient selection, and poor identification of confounding factors. It is difficult therefore, to generalise the findings of the studies to the populations from which the study participants were derived. Furthermore, it is unknown whether participants were representative of the population from which they were recruited. As such, all five studies performed poorly on the external validity questions, scoring a mean of only 27% for questions 11 to 13 on the quality Index tool.

All studies also rated poorly on the internal validity component of the Quality Index (questions 14 to 26). For example, Moretti *et al.* [26] conducted a randomised controlled study in participants with neuropathic diabetic foot ulcers, to evaluate standard care and ESWT (intervention group) against standard care alone (control group). While it is unclear what standard care comprised of in both groups, it appears that both groups received therapeutic footwear, debridement and dressings, and treatment of infection where present. Subsequently, treatment effects in the intervention group could have been influenced by the variation in the standard care regime, especially where antibiotics were used to treat infection. In the study by Saggini *et al.* [27], the authors described the control group as receiving usual conservative dressings but did not clearly describe the standard care received by the intervention group. The lack of standardization is of particular concern considering the non-blinded design of the studies. Therefore, the conclusions made by the authors of these studies should be considered in light of these methodological flaws.

The internal validity of the studies identified may also have been threatened due to a loss of participants during the study. While Moretti *et al.* [26] and Saggini *et al.* [27] suggested that all participants in their study completed the respective trials; Wang *et al.* [28] described a loss of four participants during their study, with no reference to how these losses were accounted for in their final analysis. In their second study, Wang *et al.* [29] described a loss of 11 participants during their trial, again with no discussion or statistical analysis to account for this loss. As there was no reference to an intention-to-treat analysis in the studies by Wang *et al.* [28,29], group characteristics may have changed during the trial, resulting in over-estimation of the treatment effect; only the study by Schaden *et al.* [17] made an attempt to adjust their statistical analysis with intention-to-treat analysis. Further threats to internal validity might have occurred as no attempt was made to blind those responsible for measuring the outcomes in any of the five studies. Furthermore, the details of randomization in the study by Moretti *et al.* [26] were not made clear in their study. Consequently, differences in baseline characteristics may have influenced the effects of the intervention.

The classification of ulceration varied across the five studies identified in this review. Moretti *et al.* [26] defined their inclusion of neuropathic diabetic foot ulceration as occuring below the malleoli for a period of at least 6 months with an area wider than 1 cm^2^. Although Moretti *et al*. [26] made an attempt to define peripheral neuropathy and standard care, ulcer classification and measurement of change in ulcer size was not undertaken using a recognised or validated measure. Saggini *et al.* [27] included venous and diabetic ulcers in those participants with a history of chronic ulcers for more than three months. While Saggini *et al.* [27] defined a recognised measure of wound exudate, the measurement of change in ulcer size was not appropriately described in their methods. The two studies by Wang *et al.* [28,29] included participants with recurrent chronic diabetic ulcers of the foot for more than three months duration. In these two studies [28,29], and in the study by Schaden *et al.* [17], there was no description of a recognised method used to determine the change in ulcer size following the intervention. Consequently, the variation in ulcer classification and the poor definitions of change in ulcer size used across studies renders these results susceptible to bias.

The ESWT protocol varied between studies resulting in study heterogeneity, making comparison difficult. Specifically, there were differences in the duration, frequency and strength of ESWT application identified between studies. Furthermore, these differences were also noticeable within studies. For example, a repeat course of treatment was performed in cases with incomplete healing from the first course of treatment in the study by Wang *et al.* [28]. Moreover, the ESWT treatment dosage for each participant in the studies by Wang *et al.* [29] and Schaden *et al*. [17] was dependent on the size of the participant’s ulcer, rather than a pre-determined intervention dose. In the study by Saggini *et al.* [26], participants’ received anywhere between four and 10 sessions of ESWT; essentially, in these three studies, the likelihood of the results being due to the actual intervention cannot be determined.

This systematic review has identified a number of important implications for future research. Firstly, to reduce bias it is essential that when evaluating the effectiveness of ESWT for the treatment of lower limb ulceration, that rigorous randomised controlled trial (RCT) methods are used [30,31]. Second, it is necessary that outcome measures used are reliable and valid and include both specific and generic measures [32]. This should also include detailed information about the criteria used to identify the presence of lower limb ulceration as there is substantial variability in the criteria used. Third, acknowledgement and adjustment for confounding variables should be included in future trials and if necessary, stratification of analyses should be made on the basis of the type of ulceration. This will ensure that such trials include sufficient information for the methods to be critiqued and allow comparisons to be made with similar investigations. Finally, the establishment of an optimal ESWT regimen remains to be established and should be the focus of future research.

The existing evidence that supports the use of ESWT for treatment of lower limb ulceration therefore needs to be viewed in light of some limitations. Firstly, there were only two studies (one of which was an RCT) that investigated the effect of ESWT versus standard treatment, and there were small participant numbers in the studies identified. Secondly, this review identified significant methodological heterogeneity between studies. For example; one of the studies in this review included smokers and also assessed ulceration associated with multiple comorbidities [27], whereas the other three studies did not. Third, the outcome measures assessed across studies was inconsistent; although all studies assessed the change in ulcer size or ulcer healing rates as their primary outcome, the definitions used to determine these changes varied between studies. There were some limitations to our review design; there was no pooling of data for meta-analysis and no statistical measure of heterogeneity performed. There were however, two strengths of this review; the use of a validated quality assessment tool [20], and the systematic approach used.

## Conclusions

This systematic review identified five studies that reported on the effectiveness of ESWT for the treatment of lower limb ulcers. There is limited evidence to support ESWT as a treatment for lower limb ulceration. Considering this, further research is needed to support the use of ESWT in the treatment of lower limb ulceration.

## Additional files

Additional file 1:
**Data extraction rorm.** Contains a copy of the form used to extract data from the studies included in this systematic review. Additional file 2:
**Search results by database.** Contains the results of the electronic database search for this systematic review. Additional file 3:
**Included studies.** Contains a list of included studies in this systematic review. Additional file 4:
**Reasons for the exclusion of studies after full text assessment.** Contains a list of studies excluded from this systematic review.
