# Supplementary material for: The effectiveness of extracorporeal shock wave therapy for the treatment of lower limb ulceration: a systematic review
Source: J Foot Ankle Res. 2015 Feb 5;8:3. doi: 10.1186/s13047-014-0059-0 (PMC4342213; doi:10.1186/s13047-014-0059-0)
Supplement: Additional file 1: — Data extraction rorm. Contains a copy of the form used to extract data from the studies included in this systematic review. [file 13047_2014_59_MOESM1_ESM.docx]

**Additional Data File 1: Data Extraction Form**

**Paper number**: __________________________________________________________________________________

**Included/ Excluded and reason:**

Inclusion criteria: *English language, peer reviewed, over 18 years, lower limb ulceration*

Exclusion criteria: *burn wounds, ulceration due to surgical complication, case study* _________________________________________________________________________________

**Study Design:** _____________________________________________________________________

**Subject Details:**

Inclusion Criteria ___________________________________________________________________

Exclusion Criteria ___________________________________________________________________

Recruitment Procedures: ______________________________________________________________

Sample Size: _______________________________________________________________________

Sample Sex: _______________________________________________________________________

Sample Age (mean and range):_________________________________________________________

Main outcome measures: ______________________________________________________________

**Description of ulceration and ESWT method:**

**Statistical results:**
