# Supplementary material for: The effectiveness of extracorporeal shock wave therapy for the treatment of lower limb ulceration: a systematic review
Source: J Foot Ankle Res. 2015 Feb 5;8:3. doi: 10.1186/s13047-014-0059-0 (PMC4342213; doi:10.1186/s13047-014-0059-0)
Supplement: Additional file 2: — Search results by database. Contains the results of the electronic database search for this systematic review. [file 13047_2014_59_MOESM2_ESM.docx]

**Additional Data File 2: Search results by database**

| Medline | 125 |
| --- | --- |
| CINAHL | 40 |
| Web of Knowledge | 214 |
| SCOPUS | 145 |
| AMED | 31 |
| Total | 555 |
| **Total Without Duplication** | **123** |
