# Supplementary material for: The effectiveness of extracorporeal shock wave therapy for the treatment of lower limb ulceration: a systematic review
Source: J Foot Ankle Res. 2015 Feb 5;8:3. doi: 10.1186/s13047-014-0059-0 (PMC4342213; doi:10.1186/s13047-014-0059-0)
Supplement: Additional file 3: — Included studies. Contains a list of included studies in this systematic review. [file 13047_2014_59_MOESM3_ESM.docx]

**Additional Data File 3: Included studies**

| Principal author | | Title | Year | Volume and page numbers | *Journal* |
| --- | --- | --- | --- | --- | --- |
| Moretti  Saggini  Wang  Wang  Schaden | The management of neuropathic ulcers of the foot in diabetes by shock wave therapy  Extracorporeal shock wave therapy for management of chronic ulcers in the lower extremities  Extracorporeal shockwave treatment for chronic diabetic foot ulcers  Treatment of diabetic foot ulcers: A comparative study of extracorporeal shockwave therapy and hyperbaric oxygen therapy  Shock Wave Therapy for Acute and Chronic Soft Tissue Wounds: A Feasibility Study | | 2009  2008  2009  2011  2007 | **10**(1):54  **34**(8):1261-71  **152**(1):96-103  **92**(2):187-93  **143**(1):1-12 | *BMC Musculoskeletal Disorders*  *Ultrasound in Medicine & Biology*  *Journal of Surgical Research*  *Diabetes Research and Clinical Practice*  *Journal of Surgical Research* |
|  | |  |  |  |  |
