# Supplementary material for: The effectiveness of extracorporeal shock wave therapy for the treatment of lower limb ulceration: a systematic review
Source: J Foot Ankle Res. 2015 Feb 5;8:3. doi: 10.1186/s13047-014-0059-0 (PMC4342213; doi:10.1186/s13047-014-0059-0)
Supplement: Additional file 4: — Reasons for the exclusion of studies after full text assessment. Contains a list of studies excluded from this systematic review. [file 13047_2014_59_MOESM4_ESM.docx]

**Additional Data File 4: Reasons for the exclusion of studies after full text assessment**

| **Principal author** | **Year** | **Reason For Exclusion** |
| --- | --- | --- |
| Antonic | 2011 | Review only |
| Arno | 2010 | Assessment of burns |
| Chen | 2013 | Assessment of infection associated with surgical complication |
| Ciccone | 2012 | Did not assess ulceration |
| Ennis | 2011 | Review only |
| Fioramonti | 2012 | Case report |
| Franco | 2013 | Case report |
| Ito | 2011 | Did not assess ulceration |
| Jankovic | 2011 | Case report |
| Mittermayr | 2012 | Review only |
| Moon | 2013 | Did not assess ulceration |
| Qureshi | 2011 | Review only |
| Santos | 2007 | Data not presented in a peer reviewed journal |
| Serizawa | 2012 | Did not assess ulceration |
| Serizawa | 2010 | Did not assess ulceration |
| Tse | 2011 | Did not assess ulceration |
| Wukich  Saggini  Larking  Wolff | 2010  2013  2010  2011 | Review only  Included assessment of burns  Included assessment of ulceration above the lower limb  Included assessment of burns and surgical complications |
